# Tumour cell apoptosis modulates the colorectal cancer immune microenvironment via interleukin-8-dependent neutrophil recruitment

**DOI:** 10.1038/s41419-022-04585-3

**Published:** 2022-02-04

**Authors:** Vanessa Schimek, Katharina Strasser, Andrea Beer, Samantha Göber, Natalie Walterskirchen, Christine Brostjan, Catharina Müller, Thomas Bachleitner-Hofmann, Michael Bergmann, Helmut Dolznig, Rudolf Oehler

**Affiliations:** 1 Department of General Surgery, Division of Visceral Surgery, Medical University of Vienna, Waehringer Guertel 18-20, 1090 Vienna, Austria; 2 Department of Pathology, Medical University of Vienna, Waehringer Guertel 18-20, 1090 Vienna, Austria; 3 Department of General Surgery, Division of Vascular Surgery, Medical University of Vienna, Waehringer Guertel 18-20, 1090 Vienna, Austria; 4 Institute of Medical Genetics, Medical University of Vienna, Waehringer Straße 10, 1090 Vienna, Austria

**Keywords:** Cancer microenvironment, Cell death and immune response

## Abstract

Sporadic apoptosis of tumour cells is a commonly observed feature of colorectal cancer (CRC) and strongly correlates with adverse patient prognosis. The uptake of apoptotic cell debris by neutrophils induces a non-inflammatory, pro-regenerative, and hence potentially pro-tumorigenic phenotype. In this study, we therefore sought to investigate the impact of apoptotic CRC cells on neutrophils and its consequence on other immune cells of the tumour microenvironment. Apoptosis induced by combined TNFα-treatment and UV-C irradiation, as well as various chemotherapeutic agents, led to a substantial release of neutrophil-attracting chemokines, most importantly interleukin-8 (IL-8), in both primary patient-derived and established CRC cells. Accordingly, conditioned media of apoptotic tumour cells selectively stimulated chemotaxis of neutrophils, but not T cells or monocytes. Notably, caspase-inhibition partially reduced IL-8 secretion, suggesting that caspase activity might be required for apoptosis-induced IL-8 release. Moreover, apoptotic tumour cell-conditioned media considerably prolonged neutrophil lifespan and induced an activated CD66b^high^CD11b^high^CD62L^low^ phenotype, comparable to that of tumour-associated neutrophils in CRC patients, as assessed by flow cytometry of dissociated CRC tissues. Immunohistochemical analyses of 35 CRC patients further revealed a preferential accumulation of neutrophils at sites of apoptotic tumour cells defined by the expression of epithelial cell-specific caspase-cleaved cytokeratin-18. The same areas were also highly infiltrated by macrophages, while T cells were virtually absent. Notably, neutrophils induced an M2-like CD86^low^CD163^+^CD206^+^ phenotype in co-cultured monocyte-derived macrophages and suppressed LPS-induced pro-inflammatory cytokine release. In an in vitro transwell model, IL-8 blockade efficiently prevented neutrophil-induced anti-inflammatory macrophage polarisation by inhibiting neutrophil migration towards IL-8 gradients generated by apoptotic CRC cells. To conclude, our data suggest that apoptotic cancer cells release chemotactic factors that attract neutrophils into the tumour, where their interaction with neighbouring macrophages might promote an immunologically unfavourable tumour microenvironment. This effect may contribute to tumour recurrence after chemotherapy-induced apoptosis.

## Introduction

Spontaneous apoptosis of tumour cells is frequently observed in colorectal cancer (CRC), presumably as a consequence of genomic instability and enhanced cellular turnover [1]. Although the apoptotic cell death programme is well-known for its anti-cancer properties, mounting evidence suggests that tumour cell apoptosis has the potential to support tumour growth, for example by stimulating compensatory proliferation of neighbouring cells [2, 3] or attracting reparative, growth-promoting macrophages into the tumour microenvironment [4]. Accordingly, tumour cell apoptosis strongly correlates with advanced cancer stage and adverse patient outcome in CRC [1], however, so far the underlying mechanisms remain elusive.

Neutrophils are the first immune cells recruited to sites of inflammation and essentially contribute to early host defence by phagocytosis of invading pathogens and release of pro-inflammatory granule contents [5]. Despite being traditionally regarded as a purely pro-inflammatory cell population, numerous studies suggest that neutrophils are functionally and phenotypically heterogeneous [6, 7]. This is exemplified by the indispensable functions of neutrophils in the orchestration of inflammation resolution and tissue repair, such as the generation of pro-resolving lipid mediators or induction of a pro-regenerative macrophage polarisation with enhanced efferocytosis capacity [8, 9]. In addition, the uptake of apoptotic cell debris by neutrophils themselves has been reported to prevent pro-inflammatory neutrophil responses including the generation of reactive oxygen species or TNFα release [10, 11], and thus likely contributes to inflammation resolution [12]. Accordingly, we could show recently that neutrophils acquire a pro-regenerative phenotype upon efferocytosis of apoptotic cell-derived extracellular vesicles (aEVs), characterised by the release of growth factors such as hepatocyte growth factor and basic fibroblast growth factor, both of which support liver regeneration after partial hepatectomy (submitted for publication). Despite being advantageous in the regenerative setting, the same growth factors have the potential to augment tumour progression [13]. Indeed, clinical studies suggest that regenerative responses induced in the liver upon partial hepatectomy promote tumour growth in patients with CRC liver metastasis [14–16], creating a clinically relevant link between tissue regeneration and cancer progression.

Considering that neutrophils efficiently efferocytose apoptotic debris, resulting in a non-inflammatory, pro-regenerative polarisation [10, 11], we hypothesised that the accumulation of cell debris as a consequence of spontaneous or therapy-induced apoptosis modulates the neutrophil phenotype in the CRC microenvironment. In the present study, we therefore used primary human tumour and immune cells to investigate the impact of tumour cell apoptosis on neutrophil chemotaxis, phenotypic alterations, and interplay with other cells of the immune tumour microenvironment. Immunohistochemical and flow cytometric analyses of human CRC specimens were additionally performed to explore the expression of neutrophil-specific and apoptosis-specific markers, as well as the neutrophil phenotype in CRC. We were able to show that apoptotic CRC cells release chemotactic factors to specifically attract neutrophils, which in turn promote an anti-inflammatory polarisation of neighbouring macrophages in the tumour microenvironment.

## Results

### Apoptotic cell-derived factors stimulate neutrophil migration, activation, and survival

**Fig. 1:**
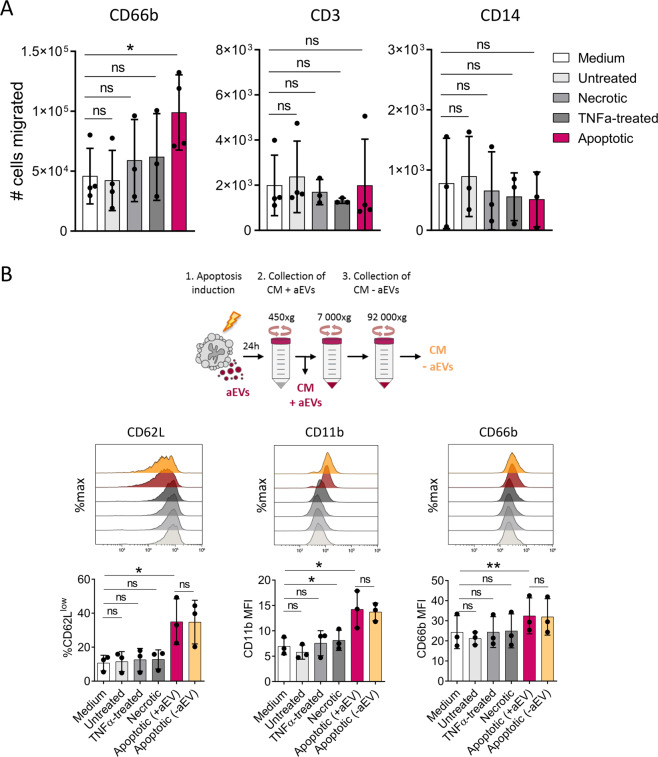
Apoptotic-tumour cell-derived factors enhance neutrophil migration and phenotypic activation. **A** Transwell migration of healthy-donor derived white blood cells towards culture medium (negative control), conditioned media (CM) of untreated or apoptotic CG08 CRC cells. Flow cytometric quantification of migrated cells was performed after 2 h using counting beads (*n* = 4). **B** Surface marker alterations of neutrophils isolated from healthy donors following stimulation for 2 h with CM of untreated, TNFα-treated, necrotic or apoptotic CG08 CRC cells, as analysed by flow cytometry (*n* = 3). Where indicated, aEVs were depleted from apoptotic CG08 CM using sequential centrifugation steps at 450 × *g*, 7000 × *g*, and 92,000 × *g* (CM-aEVs). Graphs present mean ± SD. *P < 0.05, **P < 0.01, ns not significant, as calculated by two-tailed paired *t*-tests.

To investigate whether apoptotic CRC cells promote chemoattraction of neutrophils, apoptosis was induced in primary CG08 CRC cells, which had been isolated in our laboratory from malignant ascites of a peritoneal metastatic CRC patient (for cell and patient characteristics, see Table S1). Apoptosis induction was performed by combination of TNFα treatment and subsequent UV-C irradiation, yielding approximately 75% early apoptotic cells after 24 h of treatment (Fig. S1A). In support of our hypothesis, in vitro transwell assays revealed a substantial increase in the migration of CD66b-positive neutrophils towards conditioned media (CM) of apoptotic, but not untreated, necrotic or TNFα-treated CG08 cells (Fig. 1A). In contrast, the migratory capacities of CD3-positive T cells and CD14-positive monocytes remained unchanged.

To further explore the impact of tumour cell apoptosis on neutrophil phenotype, flow cytometric analysis of isolated neutrophils stimulated with CM of apoptotic CG08 cells was performed. Indeed, a significant increase in the percentage of CD62L^low^ neutrophils could be observed, along with an upregulation of CD11b and CD66b expression (Fig. 1B). These surface marker alterations, indicative of an activated neutrophil phenotype, were not apparent in neutrophils stimulated with CM of necrotic or TNFα-treated tumour cells. Based on findings of our previous study, in which apoptotic cell-derived extracellular vesicles (aEVs) induced similar phenotypic changes in neutrophils, we assumed that aEVs in apoptotic tumour cell-CM are primarily responsible for the observed neutrophil activation. Unexpectedly however, the neutrophil activating effect remained unaltered following depletion of aEVs by ultracentrifugation (Fig. 1B). Of note, despite inducing phenotypic activation, apoptotic tumour cell-CM failed to stimulate myeloperoxidase release, which is considered a typical pro-inflammatory neutrophil response (Fig. S2).

**Fig. 2:**
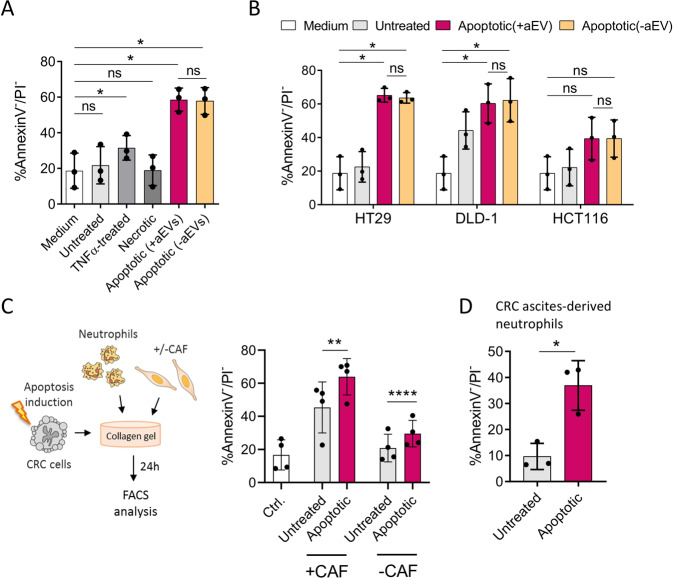
Apoptotic-tumour cell-derived factors prolong neutrophil lifespan. **A**, **B** Survival of healthy donor-derived neutrophils stimulated for 24 h with culture medium (negative control), conditioned media (CM) of untreated, TNFα-treated, necrotic or apoptotic **A** CG08, **B** HT29, DLD-1, or HCT116 cells. Neutrophil viability was determined by annexin V/propidium iodide (PI) flow cytometry staining (*n* = 3). Where indicated, apoptotic cell-derived extracellular vesicles (aEVs) were depleted from apoptotic CG08 CM using sequential centrifugation steps at 450 × *g*, 7000 × *g*, and 92,000 × *g* (CM -aEVs). **C** Survival of healthy donor-derived neutrophils in 3D CRC models. Neutrophil viability was determined by annexin V/PI staining following 24 h of co-culture with apoptotic or untreated CG08 CRC cells and cancer-associated fibroblasts (CAFs) within a collagen I matrix. Neutrophils cultured in collagen I gels in the absence of tumour cells and CAFs served as control (*n* = 4). **D** Survival of neutrophils isolated from malignant ascites of CRC patients, after 24 h stimulation with CM of untreated or apoptotic CG08 CRC cells (*n* = 3). Graphs present mean ± SD. *P < 0.05, **P < 0.01, ****P < 0.001, ns not significant, as calculated by two-tailed paired *t*-tests.

Neutrophil lifespan extension essentially contributes to the accumulation of these otherwise very short-lived cells in inflamed tissues [12] and, presumably, the tumour microenvironment. Interestingly, while CM of untreated CRC cells had no impact on neutrophil survival, apoptotic tumour cell-derived factors substantially enhanced the proportion of viable neutrophils after 24 h of culture, as determined by annexin V/propidium iodide staining (Fig. 2A) and tetrazolium salt reduction assay (Fig. S3C). Once again, the observed delay in neutrophil apoptosis was independent of aEVs, which had been depleted by ultracentrifugation. Notably, the survival-promoting effect induced by apoptotic tumour cells could be replicated using CM of three additional commercially available CRC cell lines (HT29, DLD-1, and HCT116), suggesting a general rather than a cell line-specific mechanism (Fig. 2B). To confirm the apoptotic tumour cell-mediated impact on neutrophil viability in a culture model more accurately reflecting the in vivo CRC microenvironment, neutrophils were embedded in a 3D collagen matrix together with CG08 tumour cells and cancer-associated fibroblasts. Despite cancer-associated fibroblasts had a considerable pro-survival effect on neutrophils, apoptotic CRC cells further enhanced neutrophil viability regardless of the presence of fibroblasts (Fig. 2C). To further ascertain that our findings do not only apply to healthy donor-derived peripheral blood neutrophils, neutrophils were isolated from malignant ascites of peritoneal metastatic CRC patients. The presence of tumour cells in malignant ascites makes this compartment a valuable source of extravasated neutrophils exposed to tumour-specific factors. Indeed, apoptotic tumour cell-CM significantly enhanced the survival of malignant ascites-derived neutrophils, as indicated in Fig. 2D.

### Apoptotic CRC cells release neutrophil chemotactic factors

**Fig. 3:**
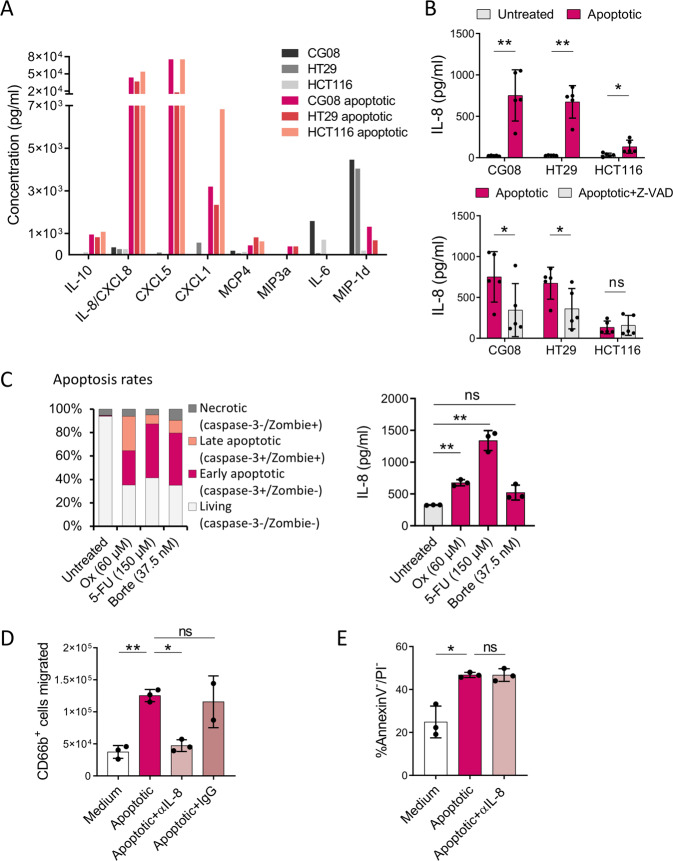
Apoptotic CRC cells promote neutrophil chemotaxis via IL-8 release. **A** Cytokine release of untreated or TNFα + UV-C treated (apoptotic) CG08, HT29 and HCT116 cells, measured in 24 h conditioned media (CM) using a multiplex cytokine panel. **B** IL-8 levels in 24 h CM of untreated or TNFα + UV-C treated (apoptotic) CG08, HT29 and HCT116 cells (upper panel), with or without 10 µM Z-VAD-FMK treatment (lower panel) (*n* = 5). **C** Apoptosis rates (left panel) and IL-8 levels (right panel) of CG08 cells treated for 72 h with 60 µM Oxaliplatin, 150 µM 5-FU or 37.5 nM Bortezomib, as analysed by flow cytometry and ELISA, respectively (*n* = 3). **D** Transwell migration of healthy-donor derived neutrophils towards culture medium (negative control) or apoptotic CG08 CM with or without 1 µg/ml anti-IL-8 (*n* = 3) or IgG control antibodies (*n* = 2). Flow cytometric quantification of migrated cells was performed after 2 h using counting beads. **E** Survival of healthy donor-derived neutrophils stimulated for 24 h with culture medium (negative control) or CM of apoptotic CG08 CRC cells with or without 1 µg/ml anti-IL-8. Neutrophil viability was determined by annexin V/propidium iodide (PI) staining (*n* = 3). Graphs present mean ± SD.*P < 0.05, **P < 0.01, ns not significant, as calculated by two-tailed paired *t*-tests.

Having shown that apoptotic CRC cells are capable of modulating neutrophil migration, phenotype and viability, presumably via the release of soluble factors, we sought to identify the responsible molecules using a 40-plex cytokine panel. Of the 40 factors analysed, eight were detectable in CM generated from three CRC cell lines (Fig. 3A). Specifically, apoptotic CG08, HT29 and HCT116 cells released elevated amounts of CXCL1, CXCL5 and interleukin-8 (IL-8), all of which are bona fide neutrophil chemoattractants. Notably, concentrations of the highly pro-inflammatory cytokine IL-6 were downregulated, while IL-10 levels concomitantly increased in apoptotic CRC cell-CM.

Since IL-8 is the most potent and neutrophil-specific chemokine among the detected factors, we assumed that it might essentially contribute to the observed neutrophil modulation induced by apoptotic tumour cells. In the following experiment, we therefore confirmed the elevated IL-8 concentrations in CM of apoptotic CRC cells by means of a more accurate IL-8 ELISA (Fig. 3B, upper panel). Treatment with the pan-caspase inhibitor Z-VAD-FMK prior to apoptosis induction, which completely prevented apoptosis as determined by absent cleaved caspase-3 staining (Fig. S1B), markedly reduced IL-8 release in CG08 and HT29 cells (Fig. 3B, lower panel). Targeting the extrinsic apoptosis pathway exclusively using the caspase-8 inhibitor Z-IETD-FMK, however, lowered IL-8 secretion to a much lesser extent (Fig. S4).

It has been previously demonstrated that depending on the apoptosis-inducing agent, patterns of cytokine release may differ [17]. We therefore aimed to investigate IL-8 release following treatment of CRC cells with additional apoptosis inducers, including Bortezomib, 5-Fluorouracil (5-FU) and Oxaliplatin, of which the latter two constitute the standard chemotherapy regimen for CRC patients [18]. As shown in Fig. 3C, primary metastatic CRC CG08 cells exhibited comparable sensitivities to all tested chemotherapeutic drugs (left panel). While treatment with Oxaliplatin and 5-FU significantly enhanced IL-8 release after 72 h, IL-8 levels only marginally increased upon Bortezomib treatment (right panel). Comparable responses to Oxaliplatin, 5-FU and Bortezomib could be observed in the CRC cell line HT29 (Fig. S5B). Interestingly, elevated IL-8 levels could already be measured 24 h after Oxaliplatin and 5-FU treatment, despite cleaved caspase-3 was only detectable in a minor percentage of cells at this time point (Fig. S5A, B), implying that IL-8 is induced as an early response to Oxaliplatin and 5-FU exposure.

To examine whether IL-8 is indeed responsible for the observed neutrophil responses induced by apoptotic CRC cells, apoptotic tumour cell-CM were treated with IL-8 blocking antibodies. IL-8 blockade almost completely abrogated neutrophil migration towards CM of apoptotic CG08 (Fig. 3D) and HT29 cells (Fig. S6), while concomitantly, neutrophil viability remained unaffected (Fig. 3E). These findings indicate that IL-8 blockade is capable of effectively preventing neutrophil migration towards apoptotic CRC cells, while factors other than IL-8 are responsible for the observed survival prolongation.

### Neutrophils co-localise with apoptotic tumour cells and macrophages in human CRC

**Fig. 4:**
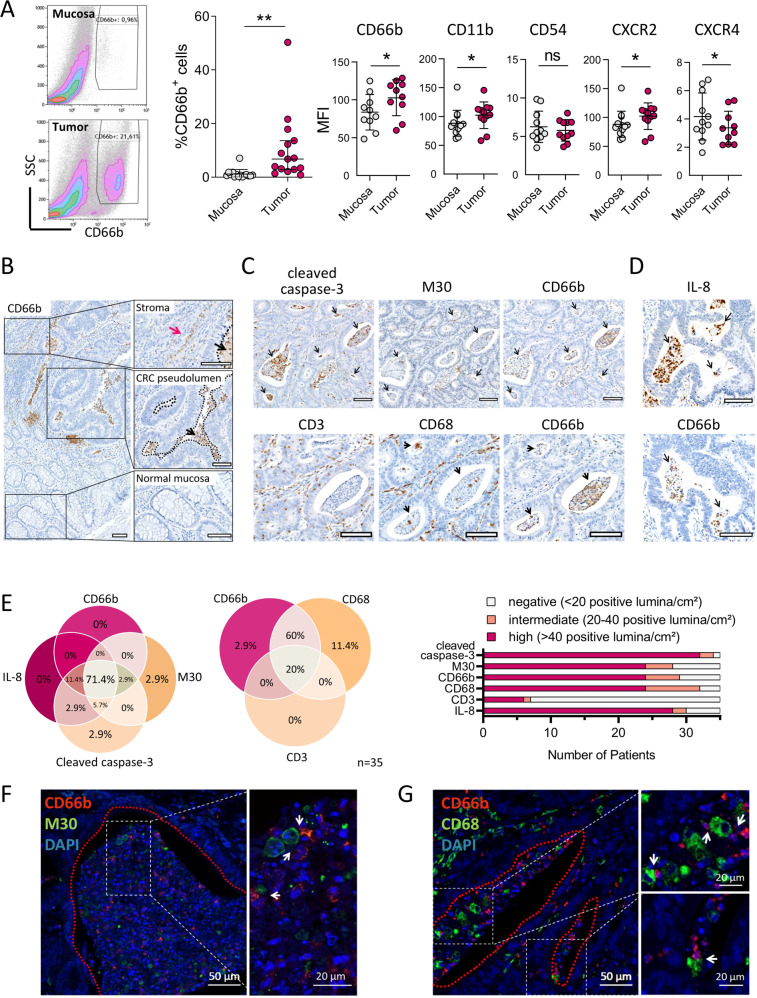
Neutrophils co-localise with apoptotic tumour cells and tumour-associated macrophages in CRC pseudolumina. **A** Flow cytometric analysis of dissociated CRC tissues and matched normal mucosae of treatment-naïve CRC patients. For gating strategy, see Fig. S7A. Graphs present median and 95% CI (%CD66b+ cells, *n* = 15) or mean ± SD (surface marker MFIs, *n* = 10–12). *P < 0.05, **P < 0.01, ns not significant, as calculated by Wilcoxon signed-rank test (%CD66b+ cells) or two-tailed paired *t*-tests (surface marker MFIs). **B** Representative CD66b immunohistochemistry (IHC) of a CRC tissue. Red and black arrows indicate stromal and intraluminal neutrophils, respectively, while dotted lines illustrate CRC pseudolumina. Scale bars, 100 µm. **C**, **D** Consecutive IHC sections of representative CRC specimens stained for M30, cleaved caspase-3 and CD66b (**C** upper panel), CD3, CD68 and CD66b (**C** lower panel) and IL-8 and CD66b (**D**). Arrows indicate positive staining in CRC pseudolumina. Scale bars, 100 µm. **E** Quantification of cleaved caspase-3-expressing, M30-expressing, IL-8-expressing, CD66b-expressing, CD68-expressing and CD3-expressing CRC pseudolumina in IHC sections of 35 CRC patients. Patients with at least 20 IHC-positive pseudolumina/cm^2^ tissue area were considered positive in the presented Venn diagrams. **F** CD66b and M30 immunofluorescence staining of a CRC pseudolumen. Arrows indicate close contact between CD66b-positive neutrophils and M30-positive apoptotic tumour cells. Images are representative of three CRC patients. **G** CD66b and CD68 immunofluorescence staining of CRC pseudolumina. Arrows indicate close contact between CD66b-positive neutrophils and CD68-positive macrophages. Images are representative of three CRC patients.

Based on our in vitro findings, suggesting a role for apoptotic tumour cells in promoting neutrophil accumulation by IL-8-mediated chemotaxis and lifespan extension, we decided to explore the situation in CRC patients (for patient characteristics, see Tables S2–3). Comparing dissociated CRC tissues and matched normal mucosae by flow cytometry, we observed a strong increase in the percentage of CD66b-positive cells within the tumour area (Fig. 4A). Similar to neutrophils stimulated with apoptotic tumour cell-CM in vitro, tumour-infiltrating neutrophils expressed higher levels of CD11b and CD66b, indicating an activated phenotype. Notably, analysis of chemokine receptor expression revealed that neutrophils in tumours, but not normal mucosae, exhibit a CXCR2^high^/CXCR4^low^ phenotype (Fig. 4A). To further assess the spatial distribution of tumour-associated neutrophils (TANs) in CRC tissues, CD66b immunohistochemistry was performed. A representative CD66b staining is shown in Fig. 4B, with tumorous tissue located right next to adjacent normal mucosa. While normal epithelial cells are arranged in a uniform pattern of round crypts, tumour cells form irregular, glandular structures encircling heterogeneous lumina, herein referred to as CRC pseudolumina. Both normal and neoplastic colonic crypts are interfused by stromal tissue. In addition to being dispersed throughout the stroma, CD66b-positive neutrophils preferentially accumulate in CRC pseudolumina. The luminal contents likely result from shedding of dying tumour cells and consequential inflammatory cell infiltration and are thus designated by pathologists as ‘dirty necrosis’ [19]. This term, however, describes the morphology of the dead cell content irrespective of underlying cell death pathway [20]. Notably, we observed prominent expression of cleaved caspase-3 in CRC pseudolumina, suggesting an integral role for apoptosis in CRC tissues (Fig. 4C). Using M30 antibodies, which specifically bind caspase-cleaved cytokeratin-18 and thus mark epithelial and tumour cell apoptosis, we could furthermore detect M30-positive apoptotic tumour cells in CRC pseudolumina (Fig. 4C).

Having observed that apoptotic CRC cells recruit neutrophils via IL-8 release in vitro, we expected positive IL-8 staining in CRC regions infiltrated by neutrophils. Indeed, CRC pseudolumina frequently stained positive for both IL-8 and CD66b (Fig. 4D), and Fisher’s exact test showed a significant association between IL-8 and CD66b positivity (*p* = 0.0014, *n* = 35), as defined by the presence of at least 20 positive pseudolumina/cm² tumour area. In a semiquantitative analysis of neutrophil-specific and apoptosis-specific markers in 35 CRC patients, 71.4% of patients were found to express CD66b, cleaved caspase-3, M30, as well as IL-8 in CRC pseudolumina (Fig. 4E). Co-localisation of neutrophils and apoptotic tumour cells could be further confirmed using CD66b and M30 immunofluorescence staining (Fig. 4F).

Since the ability of neutrophils to modulate surrounding immune cells of the tumour microenvironment has been repeatedly demonstrated [21–23], we sought to investigate whether T cells or macrophages were also present in CRC pseudolumina. While CD68-expressing macrophages were found in CRC pseudolumina of 91.4% of patients (Fig. 4E), CD3-positive T cells were primarily located in stromal and intratumoural regions. Accordingly, the majority of CRC patients (60%) stained positive for both CD66b and CD68, but not CD3 in pseudolumina (Fig. 4E). Immunofluorescence staining of CRC pseudolumina confirmed the presence of neutrophils and macrophages in close proximity to each other (Fig. 4G). Similar to primary CRC, a preferential accumulation of neutrophils and macrophages at sites of M30-positive apoptotic tumour cells could be observed in CRC liver metastases (Fig. S8). Taken together, these findings suggest that neutrophils migrate towards apoptotic tumour cells accumulating in CRC pseudoluminal structures, where they co-localise with tumour-associated macrophages.

### Neutrophils induce anti-inflammatory macrophage polarisation

**Fig. 5:**
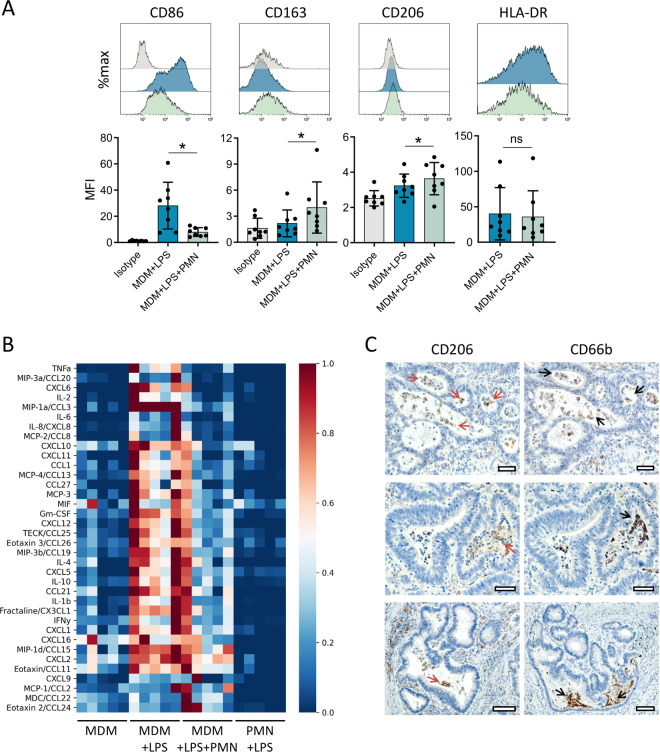
Neutrophils induce anti-inflammatory macrophage polarisation. **A** Surface marker alterations of LPS-stimulated (1 ng/ml) monocyte-derived macrophages (MDM) following 18 h of co-culture with autologous polymorphonuclear neutrophils (PMN), as analysed by flow cytometry (*n* = 8). For gating strategy, see Fig. S7B. Graphs present mean ± SD. *P < 0.05, ns not significant, as calculated by two-tailed paired *t*-tests. **B** Heatmap visualising cytokine levels in supernatants of LPS-stimulated (1 ng/ml) MDMs co-cultured for 6 h with autologous PMNs at a 1:5 ratio, as analysed using a multiplex immunoassay (*n* = 5). Cytokine concentrations were normalised to a 0–1 scale (0, blue; 1, red) and sorted by fold-change (MDM + LPS vs. MDM + LPS + PMN). **C** Consecutive IHC sections of three CRC specimens stained for CD206 and CD66b. Red and black arrows indicate positive CD206 and CD66b staining in CRC pseudolumina, respectively. Scale bars, 50 µm.

Having shown that neutrophils accumulate in CRC pseudolumina together with macrophages, we hypothesised that neutrophils might shape the tumour microenvironment via modulation of macrophage polarisation. Indeed, results of co-culture experiments‚ which were adapted from Marwick et al. [24], indicate that neutrophils polarise monocyte-derived macrophages (MDM) towards an anti-inflammatory phenotype, characterised by CD86 decrease and upregulation of CD206 and CD163 expression (Fig. 5A). In addition, neutrophils markedly impaired pro-inflammatory cytokine release by LPS-stimulated macrophages as determined in a multiplex analysis of co-culture supernatants (Fig. 5B). In support of our in vitro observations, CD206-positive, anti-inflammatory macrophages were also detectable in CRC pseudolumina in the immediate vicinity of CD66b-positive neutrophils (Fig. 5C).

**Fig. 6:**
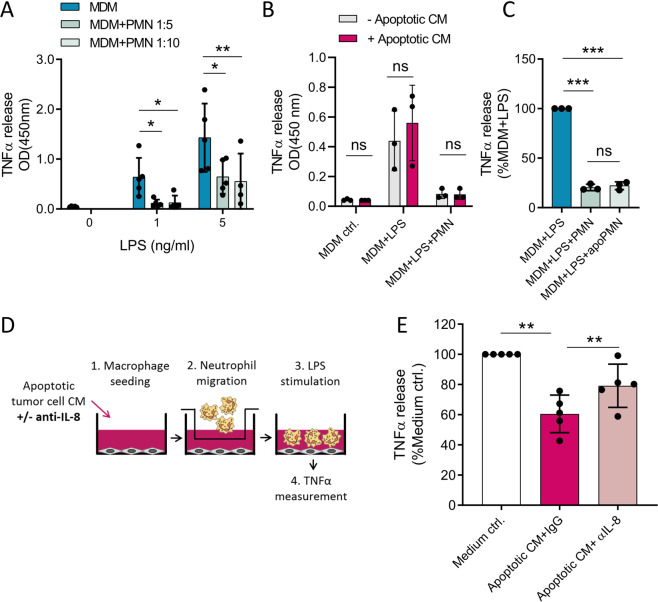
IL-8 inhibition prevents neutrophil-induced anti-inflammatory macrophage polarisation. **A** TNFα release of monocyte-derived macrophages (MDM) stimulated with indicated LPS concentrations and co-cultured for 6 h with polymorphonuclear neutrophils (PMN) at 1:5 or 1:10 ratios (*n* = 5). **B** TNFα release of MDMs stimulated with 1 ng/ml LPS and co-cultured for 6 h with PMNs at 1:5 ratios (*n* = 3). Where indicated, 50% conditioned media (CM) of apoptotic CG08 cells were added to co-cultures. **C** TNFα release of MDMs stimulated with 1 ng/ml LPS and co-cultured for 6 h with PMNs or apoptotic neutrophils (apoPMN) at 1:5 ratios (*n* = 3). **D** Schematic presentation of a modified transwell assay with MDMs seeded in the bottom chamber together with apoptotic CG08 CM with 1 µg/ml anti-IL-8 or IgG control antibodies. Isolated, autologous neutrophils are allowed to migrate for 2 h, before removal of transwells and stimulation of MDMs with 1 ng/ml LPS. TNFα release, serving as an indicator of macrophage polarisation, is measured after 18 h of culture. **E** TNFα release of MDMs cultured in a modified transwell assay as described in **D** (*n* = 5). Graphs present mean ± SD. *P < 0.05, **P < 0.01, ns not significant, as calculated by two-tailed paired *t*-tests.

Among all measured cytokines, TNFα was most considerably suppressed by neutrophils. Thus, TNFα ELISA was performed to confirm the neutrophil-mediated suppression of macrophage pro-inflammatory cytokine release (Fig. 6A). Notably, supernatants of neutrophils were sufficient to suppress TNFα release by macrophages (Fig. S9). The presence of apoptotic tumour cell-CM had no impact on the inhibitory effect of neutrophils on macrophage TNFα secretion (Fig. 6B). Moreover, co-culture with apoptotic neutrophils suppressed macrophage TNFα secretion to a similar extent (Fig. 6C). Taken together, simply the presence of neutrophils or neutrophil-derived supernatants prevents pro-inflammatory macrophage responses, irrespective of the stimulatory effects of apoptotic cell-derived factors on neutrophil activation and survival.

### IL-8 blockade prevents neutrophil-induced, anti-inflammatory macrophage polarisation

Considering that neutrophils induce an M2-like macrophage phenotype, inhibition of neutrophil accumulation in the tumour microenvironment presents a reasonable strategy to interfere with neutrophil-mediated immunosuppression. Since tumour cell apoptosis stimulates neutrophil chemoattraction in an IL-8-dependent manner (Fig. 3D), we hypothesised that IL-8 blockade, by inhibiting neutrophil migration towards apoptotic tumour cells, prevents neutrophil-induced anti-inflammatory macrophage polarisation. Using a modified transwell assay as described in Fig. 6D, we could indeed observe that TNFα release, which is an indicator of a pro-inflammatory macrophage polarisation, is significantly impaired in the presence of apoptotic tumour cell-CM (Fig. 6E). Since apoptotic tumour cell-CM per se does not affect macrophage TNFα release (Fig. 6B), this effect is likely mediated by neutrophils which are attracted towards apoptotic tumour cell-derived factors. Treatment of CM with IL-8 neutralising antibodies was sufficient to restore TNFα levels, presumably by preventing neutrophil migration and the consequential neutrophil-induced macrophage suppression (Fig. 6E).

## Discussion

While under homoeostatic conditions, apoptotic cells are rapidly cleared from tissues, M30-positive, apoptotic cancer cells are commonly detectable in CRC patients and have been linked to poor prognosis [1, 25]. In this study, we could show that apoptotic tumour cells, via the release of soluble factors, elicit multifaceted responses in neutrophils, including phenotypic activation, survival and chemotaxis, the latter of which was clearly IL-8-dependent. In line with these findings, Fadok et al. reported that Fas/CD95-induced apoptosis potently stimulates the release of neutrophil attracting chemokines, such as IL-8 and CXCL1, which the authors proposed to serve as ‘find-me’ signals to phagocytes. Moreover, using an in vivo chemotaxis model, Garg et al. observed a preferential recruitment of neutrophils towards apoptotic lung carcinoma cells treated with the immunogenic apoptosis inducer mitoxantrone, while cells undergoing tolerogenic apoptosis following tunicamycin treatment, as well as necrotic cells, failed to attract neutrophils [17]. These findings emphasise that the dichotomy between immunologically quiescent apoptosis on the one hand, and inflammatory necrosis on the other, is not absolute. Instead, the immunogenicity of apoptotic cells seems to depend, amongst others, on the cell type and the apoptosis inducing agent [17].

The immunological response to apoptosis is essentially affected by caspase activity, which is exemplified by the impaired release of the ‘find-me’ signal lysophosphatidylcholine upon caspase inhibition [26]. In the context of Fas/CD95-induced apoptosis, however, the secretion of IL-8 and other chemokines was not causally linked to caspase activity but was rather initiated in parallel to the apoptosis pathway [27]. In our study, using TNFα plus UV-C irradiation as apoptosis-inducing stimulus, we observed a partial reduction of IL-8 release in the absence of caspase activation, suggesting a possible link between caspase activity and IL-8 secretion.

However, although selective caspase-8 blockade completely abrogated apoptosis, suggesting that cell death is initiated via the extrinsic apoptosis pathway, IL-8 release was only marginally impaired in response to Z-IETD-FMK treatment (Fig. S4). Of note, caspase inhibition markedly increased the numbers of necrotic cells (Figs. S1B and S4), which could partially be explained by the induction of necroptosis (Fig. S4), a cell death pathway instigated upon disruption of caspase activity in the presence of a death receptor ligand. Since necroptotic cells have been previously shown to secrete high amounts of IL-8 [28], we assumed that the incomplete IL-8 inhibition observed following caspase blockade might be explained by necroptotic cell death. Necroptosis inhibition, however, did not diminish, but rather restored IL-8 secretion (Fig. S4).

In addition to promoting chemotaxis, CM of apoptotic CRC cells induced an activated neutrophil phenotype (CD66b^high^CD11b^high^CD62L^low^), comparable to that of TANs in CRC, (CD66b^high^CD11b^high^). TANs constitute a critical component of the tumour microenvironment with predominantly pro-tumorigenic functions [29, 30]. Mechanistically, tumour-infiltrating neutrophils have been shown to directly suppress anti-tumour T cell responses via arginase secretion [31, 32], as well as to stimulate tumour-promoting angiogenesis via vascular endothelial growth factor (VEGF) supply [33]. Accordingly, TANs are largely associated with poor patient prognosis in various tumour entities [30, 34]. In CRC, however, studies on the prognostic relevance of neutrophils remain controversial. While Governa et al. observed a favourable prognostic impact of CRC-infiltrating neutrophils [35], other studies reported an association between intratumoural neutrophils and adverse patient outcome [36, 37]. Despite these discrepancies are partly attributable to differences in the tumour stage of the study population [38], the ambiguous role of neutrophils in the tumour microenvironment requires further investigation. Comparing neutrophils within CRC tissues to those infiltrating the tumour-adjacent mucosa, we observed an upregulation of the IL-8-responsive chemokine receptor CXCR2 on TANs. Although the functional consequences of altered chemokine receptor expression remain poorly understood, infiltration of CXCR2^+^-neutrophils in murine lung tumours was accompanied by elevated expression of transforming growth factor-beta (TGF-β) and arginase-1, both of which severely compromise anti-tumour immunity [39]. Contrary to our observations, however, Governa et al. noticed a considerably lower expression of CXCR1/2 on TANs compared to neutrophils infiltrating tumour-adjacent tissue [35].

Despite neutrophils are remarkably short-lived cells with half-lives ranging from 6 to 8 h, the presence of cytokines, damage-associated molecular patterns or hypoxia may prolong the neutrophil lifespan, thereby facilitating neutrophil accumulation in inflamed tissues [40, 41]. Our data indicate that apoptotic tumour cell-derived factors substantially extend neutrophil lifespan, and thus may contribute to the observed neutrophil accumulation at sites of apoptosis in CRC. A similar survival advantage has been observed in neutrophils stimulated with CM of dissociated lung cancer tissues, which the authors attributed to high concentrations of IL-8 and granulocyte-macrophage colony-stimulating factor (GM-CSF) in these CM [42]. Despite both factors have been reported to promote neutrophil longevity [43, 44], our data indicate that neither GM-CSF, which was undetectable in apoptotic CM, nor IL-8, which seemed dispensable for neutrophil survival in IL-8 blocking experiments, are responsible for the observed apoptosis delay induced by apoptotic tumour cell-CM.

Regardless of the remarkable survival prolongation mediated by apoptotic tumour cells, we observed strong cleaved caspase-3 reactivity in areas of neutrophil accumulation in CRC tissues, exceeding by far the amount of M30-positive apoptotic tumour cells. Thus, we suggest that CRC-infiltrating neutrophils eventually undergo local apoptosis, rather than re-entering the circulation. Apoptotic neutrophils are rapidly cleared by macrophages, in which the interaction with apoptotic cells induces an anti-inflammatory, pro-reparative phenotype. Apoptotic cell clearance critically regulates inflammation resolution by terminating pro-inflammatory cytokine release and stimulating the secretion of growth-promoting and angiogenesis-promoting factors [9, 45]. At the same time, however, this process might support tumour progression [4, 46]. Less intuitively, Marwick et al. observed that not only apoptotic, but also viable neutrophils potently suppress LPS-induced pro-inflammatory macrophage responses [24], which is consistent with our findings. We additionally noted a distinct surface marker profile on macrophages co-cultured with neutrophils (CD86^low^, CD163^+^, CD206^+^), resembling an M2-like phenotype, which is generally associated with pro-tumorigenic properties [47]. Mechanistically, these effects are likely mediated by neutrophil-derived ectosomes, which have been previously demonstrated to interfere with LPS-induced, pro-inflammatory macrophage responses by inducing TGF-β secretion. The exposure of phosphatidylserine, which ectosomes share with apoptotic cells, may explain the comparable modulatory effects on the macrophage phenotype [48, 49]. Indeed, we observed that neutrophil-derived supernatants were sufficient to prevent LPS-induced pro-inflammatory cytokine release by macrophages (Fig. S9), suggesting that direct cell contact is dispensable for this effect. Contrary to this observation, however, Marwick et al. suggested that contact-dependent interaction between neutrophils and macrophages is required for the release of immunomodulatory extracellular vesicles [24].

Considering the profound impact of both apoptotic and viable neutrophils on neighbouring macrophages, we propose that therapeutic IL-8 blockade, by preventing neutrophil migration towards IL-8 gradients generated by apoptotic CRC cells, would interfere with the neutrophil-induced anti-inflammatory macrophage polarisation. Besides stimulating neutrophil chemotaxis, IL-8 exerts direct pro-tumorigenic functions, such as promoting angiogenesis, proliferation, invasion and metastasis [50]. It is therefore not surprising that high IL-8 levels are linked to poor prognosis in CRC patients [51, 52]. Interestingly, a recent study demonstrated an association between high serum IL-8 concentrations, tumour-infiltrating neutrophils and reduced benefit from immune-checkpoint blockade in various solid tumours, suggesting that IL-8 blockade might serve as a valuable complement to immune-checkpoint inhibitors by targeting a potentially unfavourable, myeloid cell-infiltrated tumour microenvironment [53]. Clinical trials evaluating the efficacy of combined IL-8 inhibition and immune-checkpoint blockade in advanced solid tumours are currently underway (NCT04572451, NCT03400332).

**Fig. 7:**
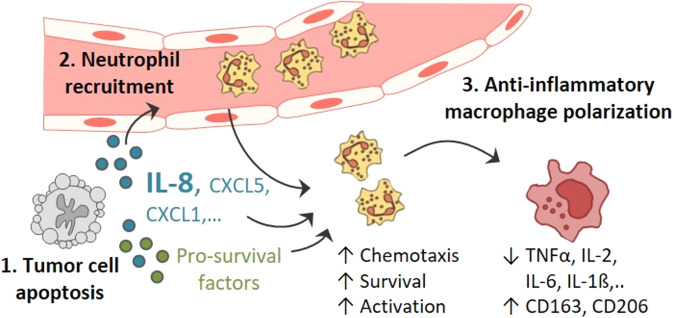
Proposed model illustrating apoptotic tumour cell-induced modulation of the CRC immune microenvironment. Apoptotic tumour cells release soluble factors, amongst others IL-8, stimulating neutrophil chemotaxis, survival and activation. Recruited neutrophils then shape the tumour microenvironment by inducing an anti-inflammatory macrophage polarisation.

In conclusion, apoptotic CRC cells induce complex functional and phenotypic responses in neutrophils, most importantly, IL-8-dependent chemotaxis. These findings are reflected by a preferential neutrophil accumulation at sites of apoptosis in CRC tissues, where these cells are found in the immediate vicinity of tumour-associated macrophages. Considering the neutrophil-induced inflammosuppressive effects on macrophages, we suggest that IL-8 blockade, by preventing neutrophil migration towards apoptotic tumour cell-derived IL-8, might present a promising strategy to therapeutically target an immunologically unfavourable tumour microenvironment in CRC (Fig. 7).

## Materials and methods

### Dissociation of colorectal cancer tissue

Primary CRC tissues and paired normal mucosae, located at least 5 cm distant from the tumour area, were resected from chemotherapy-naïve patients at the General Hospital of Vienna. All patients gave their informed consent and sample collection was approved by the ethical committee (EK Nr.: 1984/2014) of the Medical University of Vienna. Tissue dissociation was performed as described previously [54].

### Neutrophil isolation

Neutrophils were isolated from ethylenediaminetetraacetic acid (EDTA)-anticoagulated whole blood (455036, Greiner Bio-One, Kremsmünster, Austria) collected from healthy volunteers using density gradient centrifugation. Within a 15 ml tube, whole blood was layered on top of a density gradient composed of a lower layer of Histopaque 1119 (11191, Sigma-Aldrich, Vienna, Austria) and an upper layer of Ficoll-Paque PLUS (17-1440-03, GE Healthcare, Uppsala, Sweden), followed by centrifugation at 700 × *g* for 30 min. The polymorphonuclear cell fraction above the erythrocyte pellet was collected, washed with 1× Dulbecco’s phosphate-buffered saline (DPBS) (15575-020, Thermo Fisher Scientific, Vienna Austria) and resuspended in VersaLyse Lysing Solution (A09777, Beckman Coulter, Marseille, France) to remove red blood cells. The purity of isolated neutrophils ranged from 90 to 98% as determined by a haematology analyser (XN-350, Sysmex Europe GmbH, Norderstedt, Germany). To obtain neutrophils from ascites of CRC patients, ascites was collected during cytoreductive surgery of patients diagnosed with peritoneal metastatic CRC and centrifuged at 300 × *g*, followed by washing of the cells with 1× DPBS and subsequent erythrocyte lysis using VersaLyse Lysing Solution. After filtration through a 70 µm pluriStrainer (SKU 43-50070-51, Pluriselect, Leipzig, Germany), the cells were suspended in RPMI 1640 Medium (61870-044, Gibco, Thermo Fisher Scientific, Vienna, Austria) and neutrophils were isolated using the MACSxpress Whole Blood Neutrophil Isolation Kit (130-104-434, Miltenyi Biotec) according to the manufacturer’s specifications.

### PBMC and monocyte isolation

To isolate PBMCs, EDTA-anticoagulated whole blood collected from healthy volunteers was diluted with an equal volume of 1× DPBS within a 50 ml tube, followed by careful layering of Ficoll-Paque PLUS (17-1440-03, GE Healthcare) underneath the diluted blood. Centrifugation was performed at 1000 × *g* for 10 min, after which the buffy coat containing PBMCs was collected. PBMCs were washed in 1× DPBS containing 2% foetal calf serum (FCS) (SBF3111YKA, Linaris, Dossenheim, Germany) and 1 mM EDTA (15575020, Invitrogen, Paisley, UK), before monocytes were isolated using the EasySep™ Human CD14 Positive Selection Kit II (17858, Stemcell Technologies, Vancouver, Canada) in accordance with the manufacturer’s instructions.

### Cell culture

If not otherwise specified, the CRC cell lines HT29 (HTB-38, ATCC, Manassas, VA, USA), DLD-1 (CCL-221, ATCC) and HCT116 (CCL-247, ATCC), as well as the primary CRC cell line CG08, were cultured in RPMI 1640 Medium (61870-044, Gibco) supplemented with 10% FCS (SBF3111YKA, Linaris) at 37 °C in 5% CO_2_ atmosphere. All cell lines were routinely tested for mycoplasma contaminations. CG08 cells were isolated from malignant ascites of a patient diagnosed with peritoneal metastatic CRC by pathologists of the General Hospital of Vienna (for patient characteristics see Table S1). The patient material used for tumour cell isolation was obtained under informed consent (ethics committee approval EK Nr: 1756/2018) and in compliance with the EU General Data Protection Regulation. Genotypic characterisation of CG08 cells was performed by whole exome sequencing at CeGaT GmbH and is available upon request. Primary CRC-derived cancer-associated fibroblasts (CAFs) were kindly provided by Helmut Dolznig and cultured in EGM™ 2 MV medium (CC-3202, Lonza, Basel, Switzerland).

### Apoptosis induction and collection of apoptotic tumour cell-conditioned medium (CM)

One day before apoptosis induction, CRC cell lines were seeded in 12-well plates at a density of 0.4 × 10^6^ cells per well in 2 ml culture medium. Apoptosis was induced by treatment with 100 ng/ml TNFα for 1 h, followed by washing of the cell layer with 1xDPBS, addition of 2 ml fresh RPMI 1640 medium supplemented with 10% FCS per well and subsequent UV-C irradiation at 250 mJ/cm² in a UV-C chamber (CL-1000 Ultraviolet Crosslinker, VWR, Vienna, Austria). Conditioned media were collected 24 h after apoptosis induction by centrifugation at 450 × *g* or three sequential centrifugation steps at 450 × *g*, 7000 × *g*, and 92,000 × *g* to remove apoptotic cell remnants and apoptotic cell-derived extracellular vesicles (aEVs), respectively. In indicated experiments, apoptosis and/or necroptosis was blocked by pre-treating cells with 10 µM Z-VAD-FMK (N-1510, Bachem, Bubendorf, Switzerland), 50 µM Z-IETD-FMK (HY-101296, MedChemExpress, Monmouth Junction, NJ, USA) or 50 µM Necrostatin-1 (HY-15760, MedChemExpress) for one hour at 37 °C. Alternatively, apoptosis was induced by treatment of CRC cells with 60 µM Oxaliplatin (Fresenius Kabi, Lake Zurich, IL, USA), 150 µM 5-Fluorouracil (5-FU) (F6627, Sigma-Aldrich) or 37.5 nM Bortezomib (Janssen-Cilag, Beerse, Belgium) in 2 ml RPMI 1640 medium supplemented with 10% FCS. Conditioned media were collected 24 and 72 h after treatment.

### Neutrophil stimulation with apoptotic tumour cell-CM

Isolated neutrophils were suspended in RPMI 1640 medium supplemented with 10% FCS at 1 × 10^6^ cells/ml and stimulated with 50% apoptotic tumour cell-CM at 37 °C. Neutrophils were collected after 2 or 24 h for flow cytometric analysis of cell surface markers and viability, respectively. Metabolic activity of neutrophils was assessed using the EZ4U Cell Proliferation and Cytotoxicity Assay (BI-5000, Biomedica, Vienna, Austria). Briefly, 5 × 10^4^ neutrophils were cultured in 96-well plates in the presence or absence of 50% apoptotic tumour cell-CM for 24 h, followed by the addition of the EZ4U substrate according to manufacturer’s instructions. After 4 h of incubation at 37 °C, optical densities were analysed on a Varioskan LUX plate reader (Thermo Fisher Scientific). To investigate myeloperoxidase (MPO) release, 1 × 10^5^ neutrophils were suspended in 150 µl Hanks’ Balanced Salt Solution (14025-050, Gibco) and stimulated with an equal volume of FCS-free apoptotic tumour cell-CM for one hour at 37 °C. Neutrophils treated with FCS-free culture medium or the calcium ionophore A23187 (C7522, Sigma) (4 µM) served as negative and positive control, respectively. Supernatants were collected by centrifugation at 450 × *g* for 10 min and analysed using the Human Myeloperoxidase Quantikine ELISA Kit (DMYE00B, R&D Systems, Abingdon, UK) according to manufacturer’s specifications.

### Neutrophil chemotaxis

Leucocyte migration towards apoptotic tumour cell-CM was analysed using a transwell assay. One day before the experiment, 5 µm-pore transwell inserts (3421, Corning, Kennebunk, ME, USA) were coated with 100 µg/ml fibronectin (FC010, Merck, Darmstadt, Germany) for 1 h at 37 °C, washed with 1xDPBS and dried in a laminar flow hood. EDTA-anticoagulated whole blood was collected from healthy volunteers and incubated with three times the volume of VersaLyse Lysing Solution for 15 min to remove erythrocytes. Meanwhile, 600 µl RPMI 1640 medium supplemented with 50% apoptotic tumour cell-CM were added to the bottom well. Where indicated, conditioned media were pre-treated with 1 µg/ml anti-IL-8 (MA5-23697, Thermo Fisher Scientific) or IgG control antibodies (400165, Biolegend, San Diego, CA, USA) for 20 min. Subsequently, 4 × 10^5^ leucocytes suspended in 150 µl RPMI 1640 medium were added to the top chamber and allowed to migrate for 2 h at 37 °C. Migrated cells in the bottom well were eventually counted using AccuCheck Counting Beads (PCB100, Invitrogen, Paisley, UK), along with flow cytometric immunophenotyping.

### 3D neutrophil co-culture assay

To investigate neutrophil survival in a culture setting mimicking the CRC microenvironment, a 3D collagen-based co-culture system adapted from Dolznig et al. [55] was implemented. Briefly, 300 µl of a 2 mg/ml collagen I solution were prepared for each 3D gel by mixing rat-tail collagen I (354236, Corning) with 30 µl 10 × DPBS (14200-067, Gibco) and RPMI 1640 medium supplemented with GlutaMAX and 10% FCS. After having neutralised the pH using 1 M NaOH solution, 4 × 10^4^ CG08 tumour cells, 2 × 10^5^ CAFs and 1 × 10^6^ neutrophils isolated from healthy volunteers were gently suspended in the collagen I mixture and transferred into a silicone mould with a central hole of 1.5 cm diameter. Where indicated, tumour cells were harvested immediately after apoptosis induction by TNFα-treatment plus UV-C irradiation and embedded together with untreated tumour cells at a 1:5 ratio (untreated:apoptotic) into the gels. In order to prevent shrinkage of the collagen gel by CAF-mediated contractions, a nylon mesh was submerged in the collagen I solution. The gels were allowed to polymerise for 30 min at 37 °C, before removal of the silicone mould and transfer of the collagen I gel into a 12-well-plate containing 3 ml culture medium. After 24 h of co-culture at 37 °C, collagen I gels were washed twice with 1 × DPBS and dissociated in 0.125 mg/ml collagenase B (11088807001, Sigma-Aldrich) solution for 15 min on a shaker set to 750 rpm. Collagenase activity was eventually stopped by the addition of 0.1 mM EDTA, followed by 1xTrypLE (A1217701, Thermo Fisher Scientific) treatment for 10 min at 37 °C in order to obtain single cell suspensions for flow cytometric analysis.

### Macrophage-neutrophil co-culture assay

The herein described macrophage-neutrophil co-culture assay was adapted from Marwick et al. [24]. Macrophage differentiation was induced by cultivation of isolated monocytes in RPMI 1640 medium supplemented with GlutaMAX™, 10% FCS and 100 ng/ml M-CSF (130-096-491, Miltenyi Biotec, Bergisch Gladbach, Germany) for 5 days, with medium change performed on the third day. One day before the co-culture experiment, monocyte-derived macrophages (MDMs) were harvested and re-seeded in 48-well-plates at 1 × 10^5^ cells per well. To induce a pro-inflammatory phenotype, MDMs were stimulated with 20 ng/ml interferon-gamma (INFy) (Imukin®) (Boehringer Ingelheim, Vienna, Austria). On the day of co-culture, MDMs were washed twice with 1 × DPBS, before autologous neutrophils were added at a 1:5 or 1:10 ratio (MDMs:neutrophils) in RPMI 1640 medium, without FCS and M-CSF. In indicated experiments, neutrophils were cultured for 24 h before co-culture assays in order to obtain apoptotic neutrophils. After 30 min of co-culture at 37 °C, cells were stimulated with 1 ng/ml lipopolysaccharide (LPS) (L4391, Sigma-Aldrich) and cultivation was continued for 6 or 18 h. Supernatants were harvested after 6 h by centrifugation at 450 × *g* and 7000 × *g* and frozen at −80 °C. MDM phenotype was assessed after 18 h of co-culture by flow cytometry.

#### Flow cytometry

Analysis of tumour cell death was performed following incubation of cells with Zombie Yellow dye (423103, Biolegend) and intracellular active caspase-3 staining (559565, BD Pharmingen). For immune cell phenotyping, cells were stained using the following antibodies: anti-CD62L (17-0626, eBioscience, Thermo Fisher Scientific, San Diego, CA, USA), anti-CD66b (305104, Biolegend), anti-CD11b (301310, Biolegend), anti-CD16 (MHCD1617, eBioscience), anti-CD206 (321138, Biolegend), anti-CD86 (305406, Biolegend), anti-CD14 (301814, Biolegend), anti-HLA-DR (307618, Biolegend), anti-CD163 (17-1639-42, eBioscience), anti-CD45 (48-0459-41, eBioscience), anti-CD3 (17-0037-42, eBioscience). To assess neutrophil apoptosis, cells were stained with anti-CD15 (12-0159, eBioscience), annexin V and 1 µg/ml propidium iodide (V13242, FITC Annexin V/Dead Cell Apoptosis Kit, Invitrogen,). Sample acquisition was performed on a Gallios Flow Cytometer (Beckman Coulter, Indianapolis, IN, USA) and data was analysed using the Kaluza 2.1 software (Beckman Coulter). See supplementary material for extended protocol.

### Immunohistochemistry

Formalin-fixed and paraffin-embedded tissue sections were stained with anti-CD66b (305102, Biolegend), anti-cleaved caspase-3 (559565, BD Pharmingen, Franklin Lanes, NJ, USA), M30 (12140322001, Roche, Merck, Darmstadt, Germany), anti-IL-8 (MA5-23697, Thermo Fisher Scientific), anti-CD3 (MA5-14524, Thermo Fisher Scientific), anti-CD68 (M087629-2, Dako, Agilent Technologies, Carpinteria, CA, USA) and anti-CD206 (ab64693, Abcam, Cambridge, UK) antibodies for 1 h, followed by nuclear counterstaining with Hematoxylin Gill III (1.05174.0500, Merck). See supplementary material for extended protocol.

### Quantification of immunohistochemistry staining

The number of pseudolumina positive for CD66b, M30, cleaved caspase-3, IL-8, CD68 or CD3 was evaluated in consecutive paraffin sections of 35 CRC patients. Tissue sections were scanned using the Vectra Polaris Slide Scanner (Akoya Biosciences, Marlborough, MA, United States) and analysed in QuPath software [56]. Briefly, tumour areas were measured and pseudolumina with positive staining were counted. The number of positive pseudolumina per cm² was calculated and the presence of at least 20 IHC-positive lumina/cm² was considered positive. The scanned images are available upon request.

### Immunofluorescence staining

Formalin-fixed and paraffin-embedded tissue sections were incubated with anti-CD66b (305102, Biolegend), M30 (12140322001, Roche) and anti-CD68 (M087629-2, Dako) primary antibodies, followed by secondary antibody staining using donkey anti-mouse IgG-AF555 (A31570, Thermo Fisher Scientific) and goat anti-mouse IgM-DyLight650 (SA5-10153, Thermo Fisher Scientific). Nuclei were stained with DAPI (33342, Invitrogen) and sections were visualised using a LSM700 fluorescence microscope (Zeiss, Vienna, Austria). For extended protocol, see supplementary material.

### Cytokine analysis

TNFα and IL-8 concentrations were measured using the Human TNFα DuoSet ELISA (DY210-05, R&D Systems) and Human IL-8 DuoSet ELISA (DY208-05, R&D Systems) according to manufacturer’s instructions. Plates were analysed on a Varioskan LUX plate reader (Thermo Fisher Scientific). Analysis of 40 cytokines in supernatants of CRC cells and macrophages was performed using the Bio-Plex Pro™ Human Chemokine Panel (171AK99MR2, Bio-Rad Laboratories, Hercules, CA, USA) in accordance with manufacturer’s specifications. Plates were analysed using the Luminex 200 System.

### Data analysis

Graphs present mean ± SD. Two-group comparisons were performed using two-tailed paired *t*-tests for normally distributed data sets or Wilcoxon signed-rank tests and *p*-values ≤ 0.05 were considered statistically significant, as calculated using GraphPad Prism Software v.6. All statistically compared groups showed similar variances. For heatmap construction, data was normalised to a 0–1 scale (Normalised *X* = (*X* − Minimum)/(Maximum − Minimum)) and visualised using the bioinfokit package [57] in Python v.3.9.

## Supplementary information

Reproducibility checklist

Supplementary material

## Data Availability

The data generated in this study are available from the corresponding author upon request.
