# Supplementary material for: Tumour cell apoptosis modulates the colorectal cancer immune microenvironment via interleukin-8-dependent neutrophil recruitment
Source: Cell Death Dis. 2022 Feb 4;13(2):113. doi: 10.1038/s41419-022-04585-3 (PMC8816934; doi:10.1038/s41419-022-04585-3)
Supplement: Supplementary file 2 — Supplementary material [file 41419_2022_4585_MOESM2_ESM.pdf]

*Supplementary Information*

# *Tumour cell apoptosis modulates the colorectal cancer immune microenvironment via interleukin-8-dependent neutrophil recruitment*

Vanessa Schimek[1], Katharina Strasser[1], Andrea Beer[2], Samantha Göber[1], Natalie Walterskirchen[1], Christine Brostjan[3], Catharina Müller[1], Thomas Bachleitner-Hofmann[1], Michael Bergmann[1], Helmut Dolznig[4], Rudolf Oehler[1†]

[1] Department of General Surgery, Division of Visceral Surgery, Medical University of Vienna, Waehringer Guertel 18-20, A-1090 Vienna, Austria
[2] Department of Pathology, Medical University of Vienna, Waehringer Guertel 18-20, A-1090 Vienna, Austria
[3] Department of General Surgery, Division of Vascular Surgery, Medical University of Vienna, Waehringer Guertel 18-20, A-1090 Vienna, Austria
[4] Institute of Medical Genetics, Medical University of Vienna, Waehringer Straße 10, A-1090 Vienna, Austria

**[†]*Corresponding author:***
Rudolf Oehler: e-mail: rudolf.oehler@meduniwien.ac.at; Phone: +43 1 40400 73513

**Supplementary Figures S1-S9**

**Supplementary Tables S1-S3**

**Supplementary Methods**

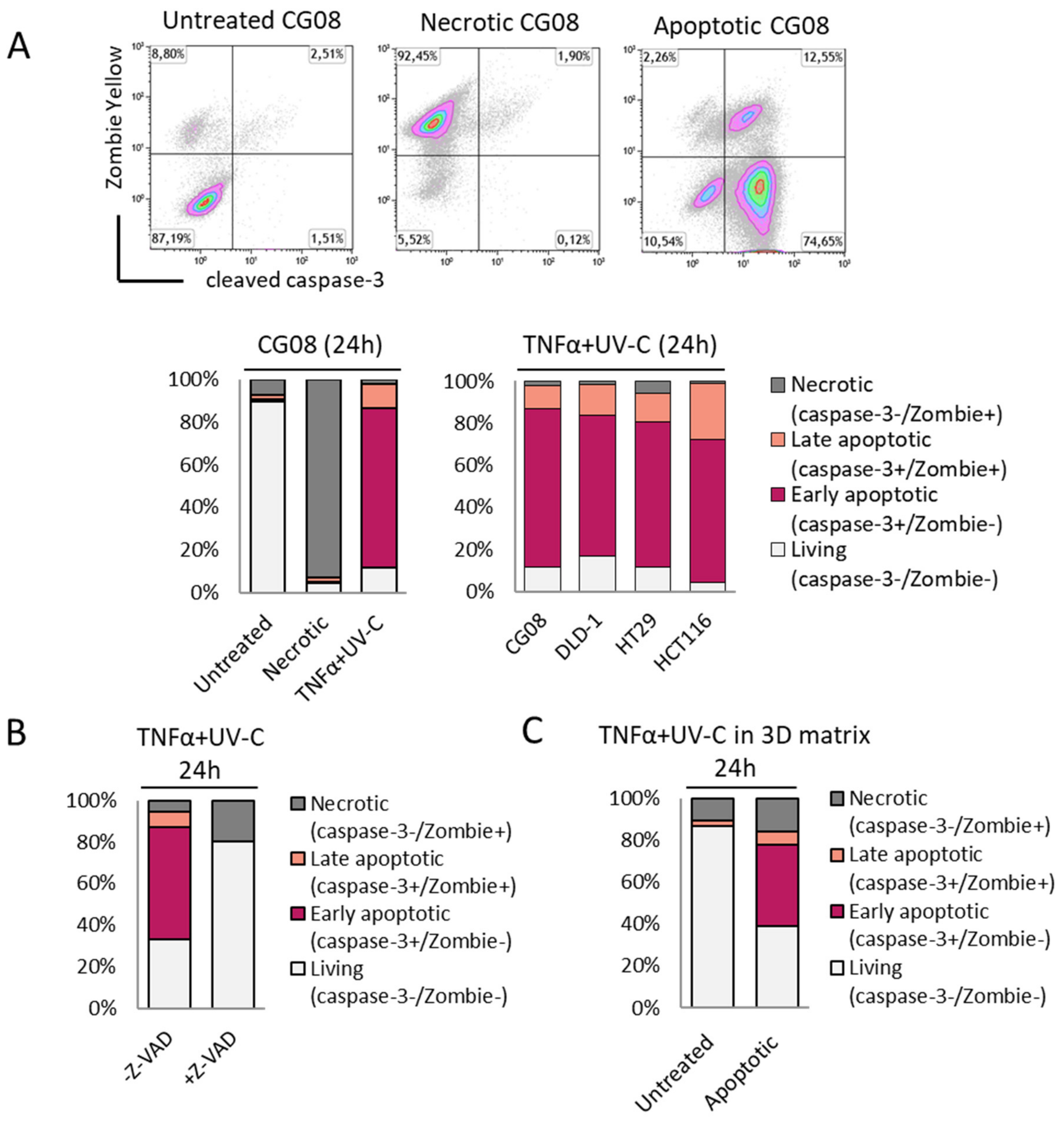


**Supplementary Figure S1: Apoptosis rates in CRC cells.** (A) Representative flow cytometry plots of untreated, necrotic and apoptotic (TNFα+UV-C-treated) CG08 cells, as determined by cleaved caspase-3/Zombie Yellow flow cytometry staining (upper panel). Percentages of living, early apoptotic, late apoptotic and necrotic CRC cells following indicated treatments are presented in the lower panel. (B) Apoptosis rates of CG08 cells treated with TNFα (100 ng/ml) and subsequent UV-C irradiation (250 mJ/cm²) in the presence or absence of Z-VAD-FMK (10 µM). Results are representative of four CRC cell lines (CG08, HT29, DLD-1, HCT116). (C) Apoptosis rates of CG08 cells after 24 hours of co-culture in a 3D collagen I matrix with cancer-associated fibroblasts and neutrophils. CG08 cells were embedded into 3D gels immediately after apoptosis induction (i.e. TNFα treatment (100 ng/ml) followed by UV-C irradiation (250 mJ/cm²)) and EpCAM staining was used to distinguish CG08 tumour cells from other cell populations within the co-culture system.

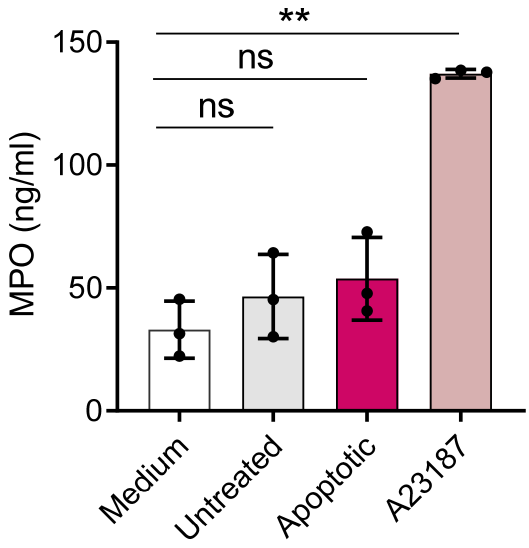


**Supplementary Figure S2: Neutrophil myeloperoxidase (MPO) release following stimulation with apoptotic CRC cell-conditioned medium.** MPO release of healthy donor-derived neutrophils stimulated for one hour with standard culture medium (negative control), conditioned medium of untreated or apoptotic (TNFα+UV-C-treated) CG08 CRC cells, or 4 µM A23187 (positive control). Apoptotic cell-derived extracellular vesicles (aEVs) were depleted from apoptotic CG08 conditioned medium using sequential centrifugation steps at 450xg, 7 000xg and 92 000xg. MPO concentrations in neutrophil supernatants were analysed by ELISA. Graphs present mean ± SD. ***P* < 0.01, ns = not significant, as calculated by two-tailed paired t-tests (n=3).

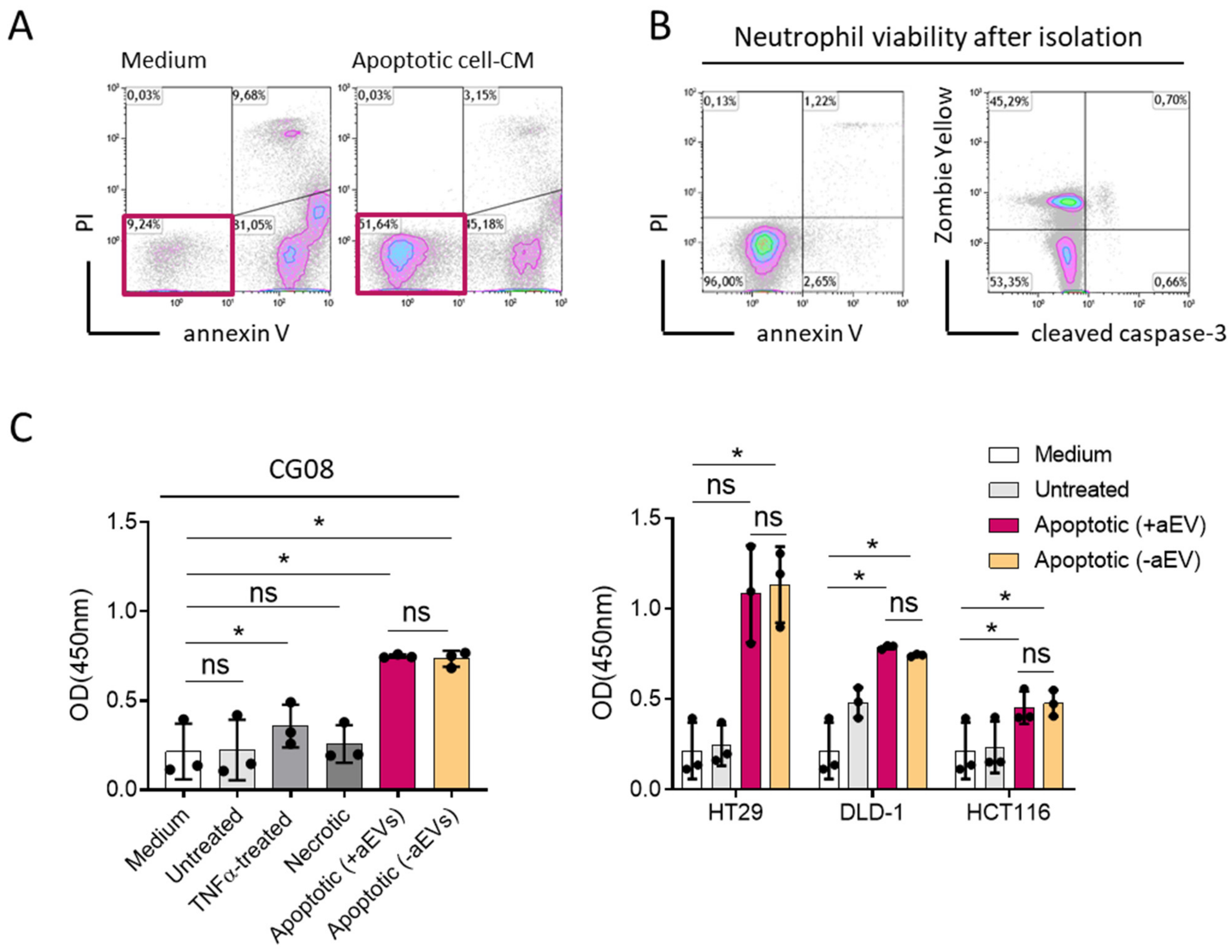


**Supplementary Figure S3: Neutrophil viability in conditioned media of apoptotic CRC cells.** (A) Representative annexin V/propidium iodide flow cytometry plots of healthy donor-derived neutrophils exposed for 24 hours to standard culture medium or conditioned medium of apoptotic (TNFα+UV-C-treated) CG08 CRC cells. Purple squares indicate viable (i.e. annexin V-/propidium iodide-negative) neutrophils. (B) Analysis of neutrophil viability immediately after isolation from peripheral blood using annexin V/propidium iodide staining (left plot) or cleaved caspase-3/Zombie Yellow staining (right plot). Note that only annexin V/propidium iodide staining correctly identifies viable neutrophils, while cleaved caspase-3/Zombie Yellow staining misclassifies freshly isolated neutrophils as Zombie Yellow-positive. (C) Neutrophil metabolic activity after 24 hours of exposure to conditioned media of untreated, TNFα-treated, necrotic or apoptotic (TNFα+UV-C-treated) CG08 (left panel) or HT29, DLD-1 and HCT116 cells (right panel). Where indicated, apoptotic cell-derived extracellular vesicles (aEVs) were depleted from apoptotic conditioned medium using sequential centrifugation steps at 450xg, 7 000xg and 92 000xg (CM -aEVs). Metabolic activity was analysed using a tetrazolium reduction assay. Graphs present mean ± SD.*$P < 0.05$, ns = not significant, as calculated by two-tailed paired t-tests (n=3).

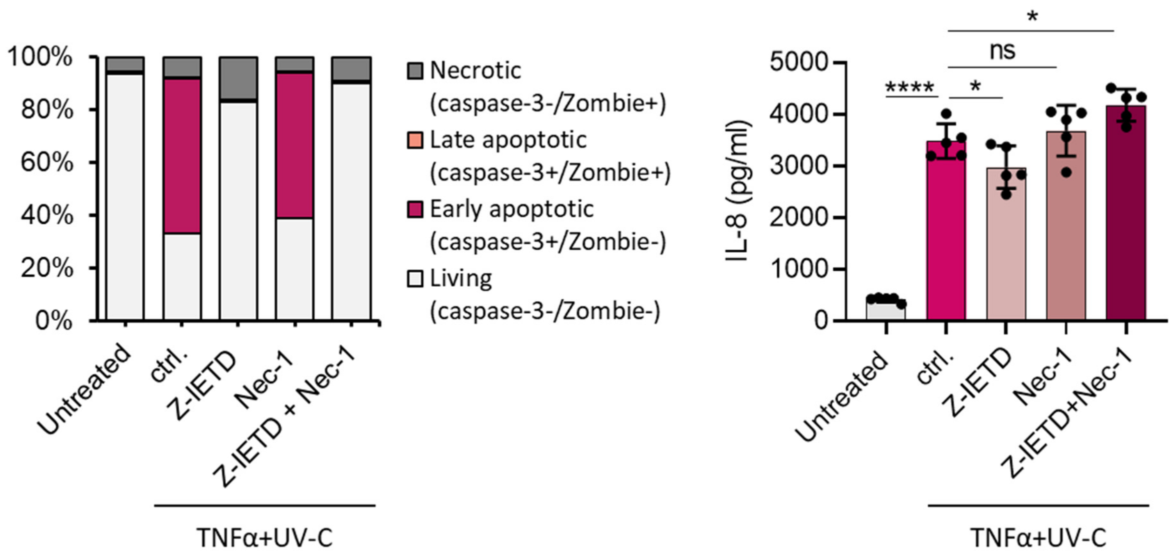


**Supplementary Figure S4: Apoptosis rates and IL-8 release in response to caspase-8 and RIPK1 inhibition.** Apoptosis rates (left panel) and IL-8 levels (right panel) of HT29 cells treated with TNFα (100 ng/ml) and subsequent UV-C irradiation (250 mJ/cm²) in the presence or absence of Z-IETD-FMK (50 µM) and/or Necrostatin-1 (Nec-1) (50 µM). Apoptosis rates were determined by cleaved caspase-3/Zombie Yellow flow cytometry staining 24 hours after treatment. Graphs present mean ± SD. *P < 0.05, ****P < 0.0001, ns = not significant, as calculated by two-tailed paired t-tests (n=5).

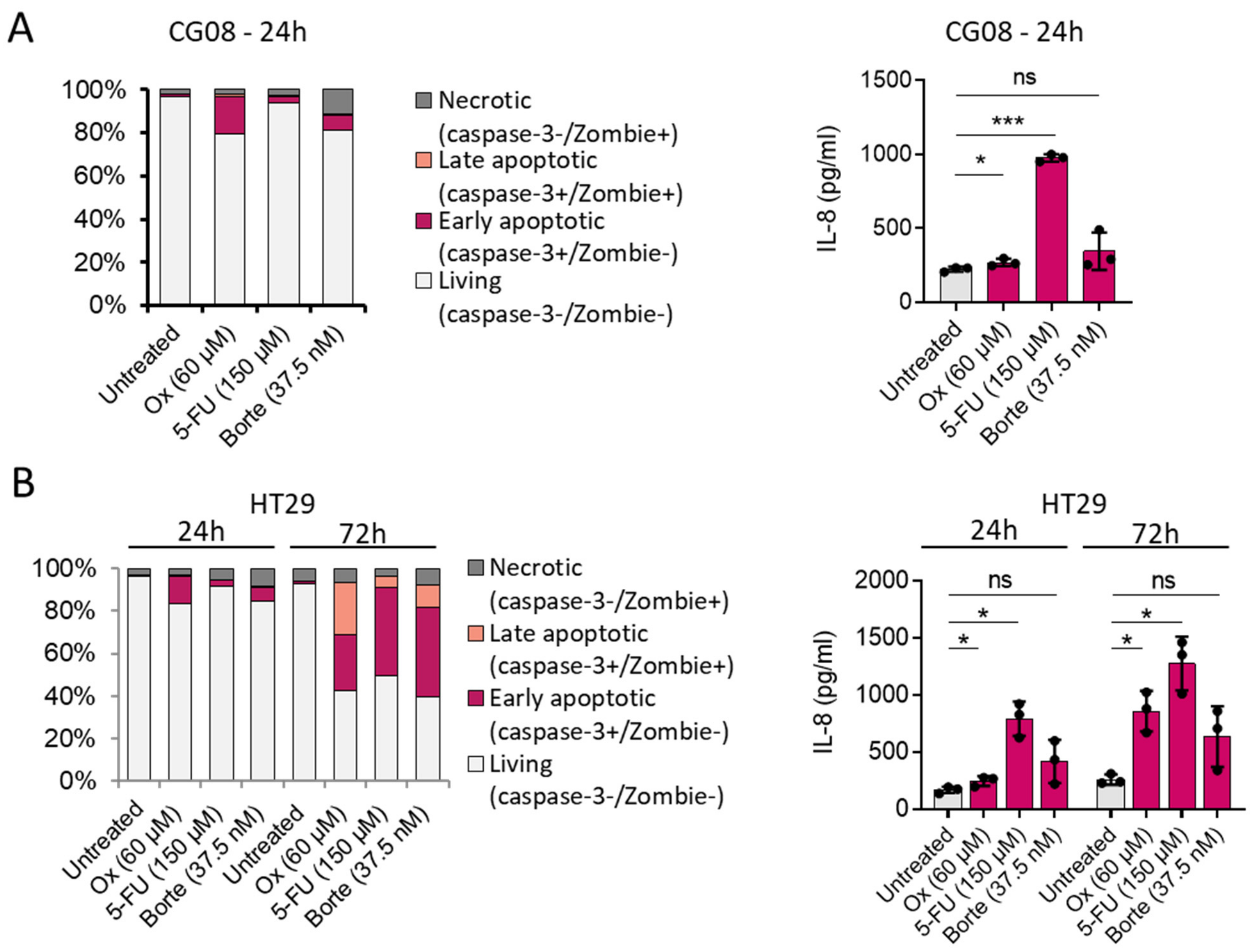


**Supplementary Figure S5: Apoptosis rates and IL-8 release in response to chemotherapy.** (A) Apoptosis rates (left panel) and IL-8 levels (right panel) of CG08 cells after 24 hours of exposure to Oxaliplatin (Ox) (60 µM), 5-Fluorouracil (5-FU) (150 µM) and Bortezomib (Borte) (37.5 nM). Apoptosis rates were determined by cleaved caspase-3/Zombie Yellow flow cytometry staining. (B) Apoptosis rates (left panel) and IL-8 levels (right panel) of HT29 cells after 24 and 72 hours of exposure to Oxaliplatin (60 µM), 5-FU (150 µM) and Bortezomib (37.5 nM). Apoptosis rates were determined by cleaved caspase-3/Zombie Yellow flow cytometry staining. Graphs present mean ± SD.**P* < 0.05, ****P* < 0.001, ns = not significant, as calculated by two-tailed paired t-tests (n=3).

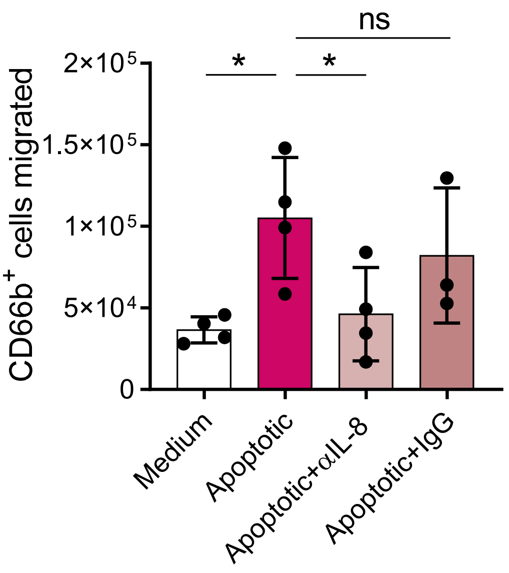


**Supplementary Figure S6: Apoptotic HT29 cells promote neutrophil chemotaxis via IL-8.** Transwell migration of healthy-donor derived neutrophils towards culture medium (negative control) or apoptotic HT29 conditioned medium with or without 1 µg/ml anti-IL-8 or IgG control antibodies. Flow cytometric quantification of migrated cells was performed after 2 hours using counting beads. Graphs present mean ± SD.**P* < 0.05, ns = not significant, as calculated by two-tailed paired t-tests (n=3-4).

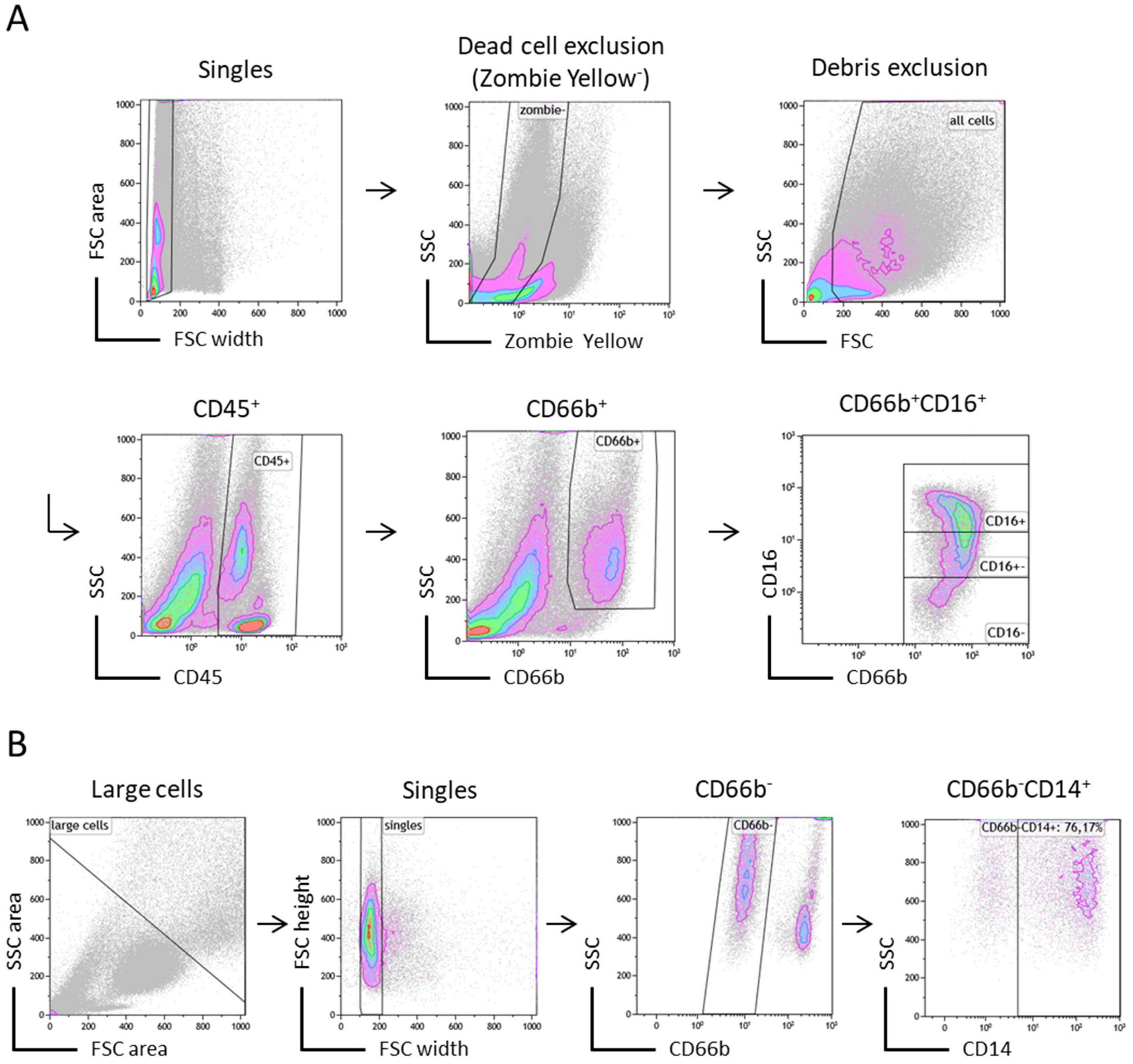


**Supplementary Figure S7: Flow cytometry gating strategies.** (A) Phenotypic characterization of human tumour-associated neutrophils: dissociated CRC and matched normal mucosae specimens were analysed by flow cytometry by gating on single, viable (Zombie Yellow-negative), CD45$^+$CD66b$^+$CD16$^+$ neutrophils. (B) Phenotypic characterization of monocyte-derived macrophages (MDMs) following *in vitro* neutrophil co-culture: after 18 hours of co-culture, MDMs were analysed by flow cytometry by gating on single, CD66b$^-$CD14$^+$ cells.

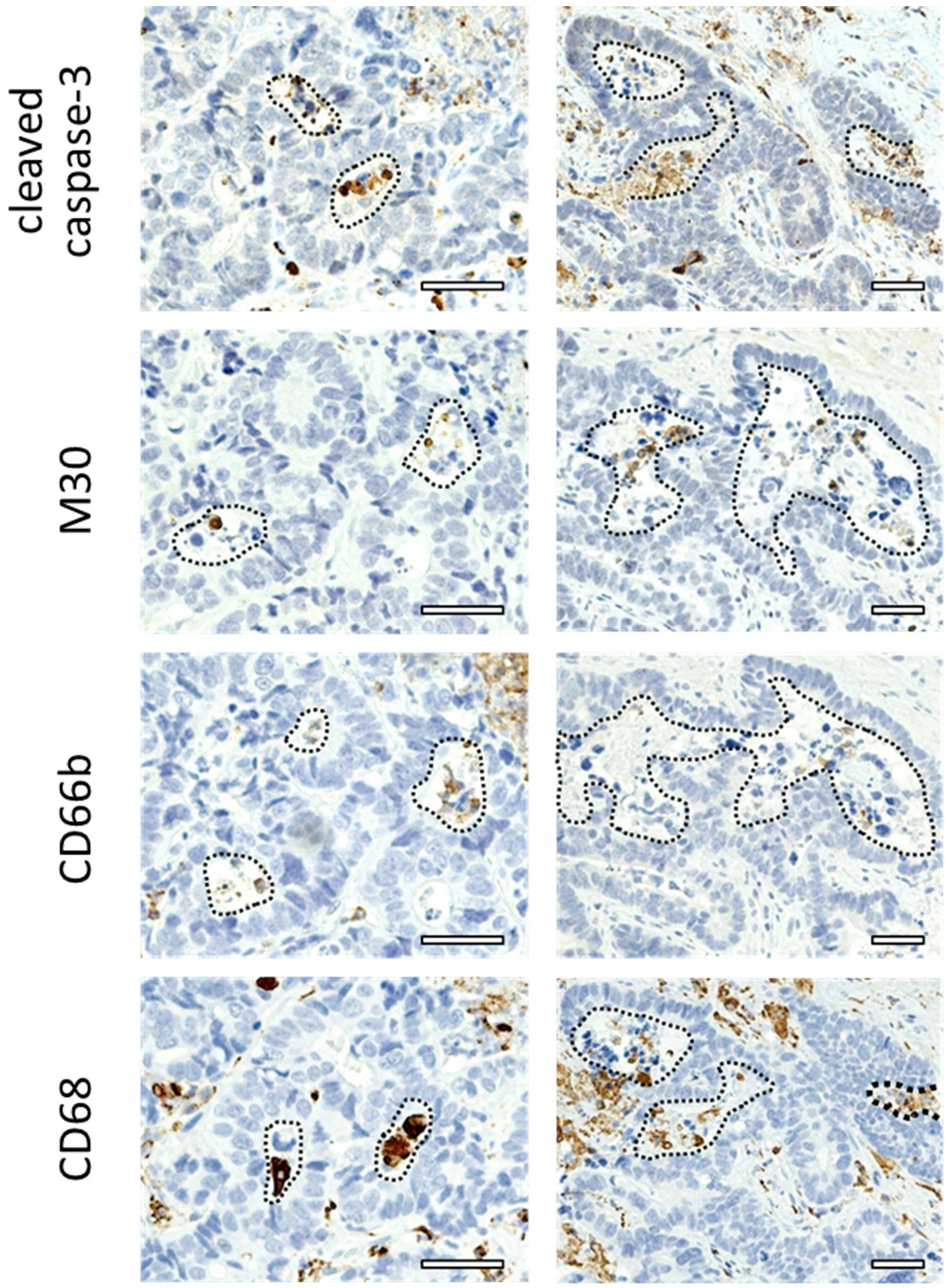


**Supplementary Figure S8: Neutrophils co-localize with apoptotic tumour cells and macrophages in CRC liver metastasis.** Consecutive immunohistochemistry sections of CRC liver metastases stained for expression of cleaved caspase-3, M30, CD66b and CD68. Dotted lines illustrate pseudolumina positive for the indicated markers. Scale bars, 50 µm.

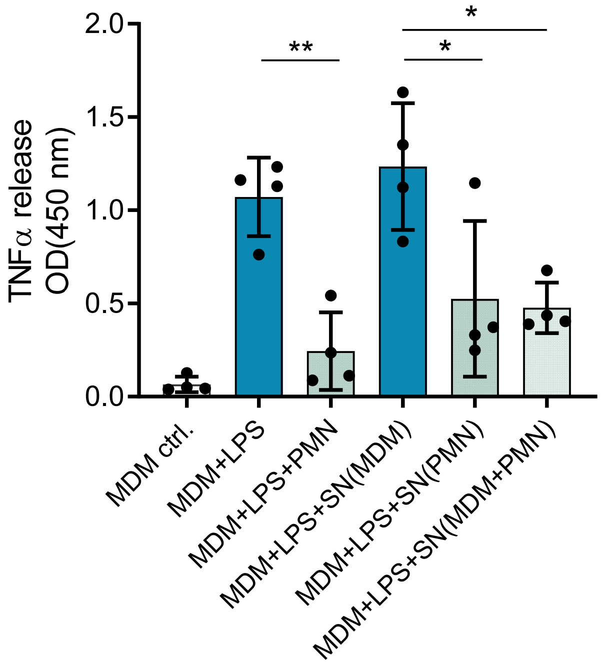


**Supplementary Figure S9: Neutrophil supernatants suppress macrophage TNFα release.** TNFα release of monocyte-derived macrophages (MDM) stimulated with 1 ng/ml LPS and co-cultured for 18 hours with polymorphonuclear neutrophils (PMN) at a 1:5 ratio, or 6-hour-supernatants (SN) of MDM, PMN or MDM+PMN co-cultures. Graphs present mean ± SD.**P* < 0.05, ***P* < 0.01, as calculated by two-tailed paired t-tests (n=4).

**Supplementary Table S1:** CG08 primary CRC cells - cell and patient characteristics

| **CG08 primary CRC cells** | |
|---|---|
| Organism | *Homo sapiens*, human |
| Source | Malignant ascites |
| Morphology | Epithelial |
| Growth properties | Adherent |
| Patient characteristics | |
| Age (years) | 19 |
| Gender | Male |
| Diagnosis | Peritoneal carcinomatosis of colorectal cancer |
| Tumour Stage (UICC) | IV (T4b, N2b, M1) |
| Microsatellite stability | Microsatellite-stable (MSS) |
| Mutation status | $TP53^{mut}$, $SMAD4^{mut}$ |

**Supplementary Table S2:** Patient characteristics - Neutrophil phenotyping

| Variables | N | % |
|---|---|---|
| Total | 16 | |
| Sex | | |
| Male | 8 | 50 |
| Female | 8 | 50 |
| Median age in years (range) | 71 (31-79) | |
| Tumour location | | |
| Colon | 13 | 81.3 |
| Rectum | 3 | 18.6 |
| Tumour stage | | |
| IIa | 6 | 37.5 |
| IIb | 1 | 6.3 |
| IIIa | 1 | 6.3 |
| IIIb | 4 | 25 |
| IVa | 2 | 12.5 |
| IVb | 2 | 12.5 |
| Neo-adjuvant chemotherapy | 0 | 0 |
| Microsatellite stability | | |
| MSS/MMR-proficient | 14 (87.5) | 87.5 |
| MSI/MMR-deficient | 2 (12.5) | 12.5 |

MSS, microsatellite stability; MMR, mismatch repair; MSI, microsatellite instability

**Supplementary Table S3:** Patient characteristics - Immunohistochemical analysis

| Variables | N | % |
|---|---|---|
| Total | 35 | |
| Sex | | |
| Male | 21 | 60 |
| Female | 14 | 40 |
| Median age in years (range) | 70 (42-82) | |
| Tumour location | | |
| Colon | 31 | 88.6 |
| Rectum | 4 | 11.4 |
| Tumour stage | | |
| I | 5 | 14.3 |
| IIa | 18 | 51.4 |
| IIc | 1 | 2.9 |
| IIIa | 1 | 2.9 |
| IIIb | 3 | 8.6 |
| IV | 7 | 20 |
| Neo-adjuvant chemotherapy | 5 | 14.3 |
| Microsatellite stability | | |
| MSS/MMR-proficient | 12 | 34.3 |
| MSI/MMR-deficient | 5 | 14.3 |
| Data not available | 18 | 51.4 |

MSS, microsatellite stability; MMR, mismatch repair; MSI, microsatellite instability

## Supplementary Methods

### *Flow cytometry*

*Tumour cell death:* Flow cytometric analysis of tumour cell death was performed by washing cells with 1xDPBS containing 2% BSA, followed by incubation with Zombie Yellow dye (423103, Biolegend, San Diego, CA, USA) diluted 1:100 in 1xDPBS for 15 minutes at 4°C. For intracellular staining of cleaved caspase-3, cells were washed with 1xDPBS containing 2% bovine serum albumin (BSA), fixed in 5.58% formaldehyde solution (F8775, Sigma-Aldrich, Vienna, Austria) for 10 minutes at 4°C, and washed again with 1x Permeabilization Buffer (00-8333-56, eBioscience, Thermo Fisher Scientific, San Diego, CA, USA). Incubation with anti-cleaved caspase-3 antibody (559565, BD Pharmingen, Franklin Lanes, NJ, USA) was performed in 1x Permeabilization Buffer for 25 minutes at 4°C. Stained cells were washed twice with 1xDPBS containing 2% BSA before measurement.

*Immune cell phenotyping:* For macrophage and neutrophil phenotyping, up to $1x10^6$ cells were washed with 1xDPBS containing 2% BSA, followed by Fc receptor blockade with human serum (P30-2901M, PAN Biotech, Aidenbach, Germany) diluted 1:2 in 1xDPBS for 10 minutes. The following antibodies were added for 25 minutes at 4°C: anti-CD62L (17-0626, eBioscience), anti-CD66b (305104, Biolegend), anti-CD11b (301310, Biolegend), anti-CD16 (MHCD1617, eBioscience), anti-CD206 (321138, Biolegend), anti-CD86 (305406, Biolegend), anti-CD14 (301814, Biolegend), anti-HLA-DR (307618, Biolegend), anti-CD163 (17-1639-42, eBioscience), anti-CD45 (48-0459-41, eBioscience), anti-CD3 (17-0037-42, eBioscience). Stained cells were washed twice with 1xDPBS containing 2% BSA before measurement.

*Neutrophil apoptosis:* To assess neutrophil apoptosis, cells were washed twice with 1xDPBS containing Ca2+ and Mg2+ (14040-091, Gibco, Thermo Fisher Scientific, Vienna, Austria) before staining with anti-CD15 (12-0159, eBioscience), annexin V and 1 µg/ml propidium iodide in 100 µl annexin-binding buffer (V13242, FITC Annexin V/Dead Cell Apoptosis Kit, Invitrogen, Paisley, UK) for 15 minutes at room temperature. Before measurement, samples were filled up with 150 µl annexin-binding buffer. It should be noted that only staining with annexin V/propidium iodide, but not cleaved caspase-3/Zombie Yellow correctly defined neutrophil viability as Zombie Yellow staining resulted in false positive signals (Figure S2B). Sample acquisition was performed on a Gallios Flow Cytometer (Beckman Coulter, Indianapolis, IN, USA) and data was analysed using the Kaluza 2.1 software (Beckman Coulter).

***Immunohistochemistry***

Formalin-fixed and paraffin-embedded tissue sections were deparaffinised in xylol (12655067, Fisher Scientific, Loughborough, UK) and rehydrated in a graded ethanol series (20821, VWR, Vienna, Austria). Endogenous peroxidases were blocked using a 0.3% hydrogen peroxide ($H_2O_2$) solution in 1xDPBS for 10 minutes at room temperature. Antigen retrieval was performed in 10 mM sodium citrate buffer (C-8532, Sigma-Aldrich) (pH 6.0) containing 0.05% Tween20 (8.22184.0500, Merck, Darmstadt, Germany) by heating to 120°C. IHC slides were blocked in Ultra V Protein Block for 5 minutes, followed by primary antibody incubation at room temperature for one hour in a humid chamber: CD66b (305102, Biolegend), cleaved caspase-3 (559565, BD Pharmingen), M30 (12140322001, Roche, Merck, Darmstadt, Germany), CD3 (MA5-14524, Thermo Fisher Scientific, Vienna, Austria), CD68 (M087629-2, Dako, Agilent Technologies, Carpinteria, CA, USA), IL-8 (MA5-23697, Thermo Fisher Scientific), CD206 (ab64693, Abcam). The UltraVision LP Large Volume Detection System (TL-060-HL, Thermo Fisher Scientific) and Liquid DAB+Substrate Chromogen System (K3468, Dako) were used for polymeric labelling and DAB 3,3′-Diaminobenzidine (DAB) staining of IHC sections, respectively. Nuclei were counterstained with Hematoxylin Gill III (1.05174.0500, Merck), followed by dehydration in a graded ethanol series. Slides were eventually collected in n-Butylacetat (1.09652.1000, Merck) and mounted with Entellan (1.07961.0100, Merck).

***Immunofluorescence***

For immunofluorescence staining, deparaffinization, rehydration and antigen retrieval were performed as described above. Slides were blocked with 1% BSA and 0.1% Tween20 in 1xDPBS for 30 minutes at room temperature, followed by primary antibody incubation over night at 4°C in a humid chamber: CD66b (305102, Biolegend), M30 (12140322001, Roche), CD68 (M087629-2, Dako). The following day, secondary antibody staining was performed for one hour at room temperature using donkey anti-mouse IgG-AF555 (A31570, Thermo Fisher Scientific) and goat anti-mouse IgM-DyLight650 (SA5-10153, Thermo Fisher Scientific) antibodies. Nuclei were stained with DAPI (33342, Invitrogen) for 10 minutes at room temperature. Stained sections were mounted with Fluoromount G (0100-01, Southern Biotech, Birmingham, AL, USA) and visualized using a LSM700 fluorescence microscope (Zeiss, Vienna, Austria).
